# Growth inhibitory and chemo-sensitization effects of naringenin, a natural flavanone purified from *Thymus vulgaris*, on human breast and colorectal cancer

**DOI:** 10.1186/s12935-015-0194-0

**Published:** 2015-04-24

**Authors:** Mohamed Salah I Abaza, Khaled Y Orabi, Ebtehal Al-Quattan, Raja’a J Al-Attiyah

**Affiliations:** Molecular Biology Program, Department of Biological Sciences, Faculty of Science, Kuwait University, Safat, 13060 Kuwait; Department of Pharmaceutical Chemistry, Faculty of Pharmacy, Kuwait University, Safat, 13110 Kuwait; Department of Microbiology and Immunology, Faculty of Medicine, Kuwait University, Safat, 13110 Kuwait

**Keywords:** Naringenin, Colorectal and breast cancers, Anticancer effects, Chemo-sensitization, Molecular mechanisms

## Abstract

**Background:**

Natural products with diverse bioactivities are becoming an important source of novel agents with medicinal potential. Cancer is a devastating disease that causes the death of millions of people each year. Thus, intense research has been conducted on several natural products to develop novel anticancer drugs.

**Methods:**

Chromatographic and spectral techniques were used for the isolation and identification of naringenin (Nar). MTT, flow cytometry, western blotting, Real Time PCR were used to test anticancer and chemosensitizing effects of Nar, cell cycle, apoptosis, and expression of cell cycle, apoptosis, pro-survival and anti-survival-related genes.

**Results:**

In the present study, *Thymus vulgaris* ethanol extract was purified repeatedly to produce several compounds including the known flavanone, Nar which was identified using different spectral techniques. Nar was shown to inhibit both human colorectal and breast cancer cell growth in a dose- and time-dependent manner through cell cycle arrest at S- and G_2_/M-phases accompanied by an increase in apoptotic cell death. Additionally, Nar altered the expression of apoptosis and cell-cycle regulatory genes by down-regulating *Cdk4, Cdk6*, *Cdk7, Bcl2, x-IAP* and *c-IAP-2* and up-regulating *p18*, *p19*, *p21*, *caspases 3*, *7*, *8* and *9*, *Bak*, *AIF* and *Bax* in both colorectal and breast cancer cells. Conversely, it diminished the expression levels of the cell survival factors PI3K, pAkt, pIκBα and NFκBp65. Moreover, Nar enhanced the sensitivity of colorectal and breast cancer cells to DNA-acting drugs.

**Discussion:**

These findings provide evidence that Nar’s pro-apoptotic and chemo-sensitizing effects are mediated by perturbation of cell cycle, upregulation of pro-apoptotic genes and down-regulation of anti-apoptotic genes and inhibition of pro-survival signaling pathways.

**Conclusion:**

In conclusion, Nar might be a promising candidate for chemoprevention and/or chemotherapy of human cancers. However, further studies exploring this therapeutic strategy are necessary.

## Background

Cancer is the leading cause of death in economically developed countries and the second leading cause of death in developing countries [1]. The burden of cancer is increasing in economically developed countries as a result of population aging and growth and, increasingly, an adoption of cancer-associated lifestyle choices, such as smoking, physical inactivity, and unhealthy food.

Colorectal cancer is the third most commonly diagnosed cancer in males and the second in females, with over 1.2 million new cancer cases and 608,700 deaths estimated to have occurred in 2008 [2]. Breast cancer is the most frequently diagnosed cancer and the leading cause of cancer death in females worldwide, accounting for 23% (1.38 million) of the total new cancer cases and 14% (458,400) of the total cancer deaths in 2008 [2].

Cancer is the second most common cause of death in Kuwait after cardiovascular diseases [3]. It has been noted that the age-adjusted incidence rates (AAIR) of colorectal cancer among Kuwaiti males has increased by approximately 5-fold over the last 33 years and ranked the most frequent site for the years 2003–2007. For Kuwaiti females, breast cancer has the highest incidence among the Kuwaiti population; breast cancer incidence has increased by 3-fold over the last 33 years. Conversely, the incidence of colorectal cancer has increased by approximately 4-fold. [3].

Cancer survival rates tend to be poorer in developing countries, most likely because of a combination of a late diagnosis and limited access to timely and standard treatment. Cancer can be treated by chemotherapy, radiation, surgery, monoclonal antibody therapy, and other methods. However, the effectiveness of chemotherapy is often limited by their serious side effects [4]. Radiation may also affect normal tissues. Therefore, complete removal of cancerous tissue without affecting normal tissue is the major goal of treatment. Approximately 50-60% of cancer patients in the United States utilize agents derived from different parts of plants or nutrients (complementary and alternative medicine), exclusively or concurrently with a traditional therapeutic regimen such as chemotherapy and/or radiation therapy. The need for new drugs has promoted studies evaluating possible anticancer agents in fruits, vegetables, herbs, and spices.

In recent years, dietary phytochemicals have received the attention of various scientists for their action in treating various cancers [5]. Several studies have documented that naturally occurring dietary phytochemicals inhibit the growth of various cancer cells through the inhibition of cell proliferation and the activation of apoptosis [6]. In particular, flavonoids, polyphenolic compounds that occur naturally in the plant kingdom, display a wide range of pharmacological properties, including antioxidant and anti-carcinogenic activities [6-9].

Thyme (*Thymus vulgaris* L., Family Lamiaceae), which is known in Arabic as “zaatar or “zaitra”, is a pleasant-smelling perennial shrub that grows in several regions worldwide [10]. The plant is indigenous to the Mediterranean region and neighboring countries, Northern Africa, and parts of Asia [11]. Thyme is widely used in folk medicine for its expectorant, antitussive, antibronchiolitis, antispasmodic, anthelmintic, carminative and diuretic properties. The aromatic and medicinal properties of the genus *Thymus* made it one of the most popular plants worldwide. *Thymus* species have strong antibacterial, antifungal, antiviral, and antioxidant activities [12]. Many pharmacological *in vitro* studies have revealed the pharmacological activities of both thyme essential oil and plant extracts [13]. Given the various uses of thyme in traditional medicine and the hypothesis that it may have anticancer activity, the present study was undertaken to fractionate *Thymus vulgarus* in a bioactivity-guided manner, to isolate and identify the bioactive lead(s) that suppress(es) colorectal and breast cancer cell growth, and to study the underlying intracellular signal transduction pathways involved in regulating cell cycle and apoptosis and its/their ability to potentiate the chemo-sensitivity of colorectal and breast cancer cells to DNA-acting drugs.

## Methods

### Cell lines

Human colorectal cancer cell lines (SW1116 and SW837), human breast cancer cell lines (HTB26, HTB132), and normal human fibroblast cells (CRL1554) were obtained from American Type Culture Collection (ATCC; VA, USA). SW1116, SW837, HTB26 and HTB132 cells were cultured in 90% Leibovitz’s L15 medium supplemented with 10% heat-inactivated fetal bovine serum and grown at 37°C in a non-CO_2_ incubator. CRL1554 cells were cultured in Eagle minimum essential medium, EMEM (90%) supplemented with 10% heat-inactivated fetal bovine serum and grown at 37°C in the presence of 5% CO_2_ and 95% ambient air.

### Chemicals and reagents

Trypsin, Leibovitz's L-15 and EMEM medium, fetal bovine serum (FBS), and penicillin/ streptomycin solution (100×) were obtained from Mediatech, Inc. (Herndon, VA, USA). An Annexin V-FITC apoptosis detection kit was obtained from BD Hoffmann-La Roche Inc. (Nutley, NJ, USA). A DNA-prep kit was obtained from Beckman & Coulter (FL, USA). All reagents for RT-PCR and real-time qPCR were obtained from Applied Biosystem (Foster City, CA, USA). Nuclear/cytosol fractionation kit was obtained from BioVision, Inc. (Moutain View, CA, USA). Antibodies against PI3K, phospho-Akt1/2/3 (Ser473), Akt, NFκBp65, pIκBα and β-actin were purchased from Santa Cruz Biotechnology (Santa Cruz, CA and Cambridge, UK). All other reagents were purchased from Sigma Chemicals (St Louis, MO, USA). Plasticware was purchased from Falcon Lab (Franklin Lakes, NJ, USA).

### General experimental procedure

Melting points were determined in open capillary tubes using a Mettler 9100 electrothermal melting point apparatus and were uncorrected. IR spectra were recorded using a JASCO FTIR-4100 spectrophotometer. UV spectra were measured in MeOH using a UV-160 IPC UV-visible dual-beam spectrophotometer. The ^1^H and ^13^C NMR spectra were obtained on a Bruker Advance II 600-MHz spectrometer operating at 600 and 150 MHz, respectively. Both ^1^H and ^13^C NMR spectra were recorded in methanol-*d*_4_, and the chemical shift values were expressed in δ (ppm) relative to the internal standard TMS. For the ^13^C NMR spectra, spectral editing was determined by DEPT. 2D NMR data were obtained using the standard pulse sequence of the Bruker 600 for COSY, HSQC and HMBC. Low resolution EIMS were obtained using a double-focusing magnetic sector mass spectrometer (GS-MS DFS/Thermo).

### Plant material

*Thymus vulgaris* was obtained commercially from the local market. Its identity was established as *Thymus vulgaris* by Dr. KT Mathew of Kuwait University. A voucher specimen was deposited at Kuwait University Herbarium and given the number KTM & IYQ (5920).

### Extraction and isolation

The dried ground plant (1.0 kg) was percolated at room temperature with 96% EtOH (1 L × 3), and the extract was evaporated *in vacuo* to leave 43 g of residue. Part of this crude extract (10 g) was partitioned between water and ethyl acetate. Part of the ethyl acetate fraction (2 g) was chromatographed over a Si gel (190 g, 38 cm × 4 cm), using 15% acetone in chloroform as the eluent, to yield, after crystallization, 5.3 mg (0.01% yield) of pure Nar as pale yellow needles. This process was scaled up to yield Nar quantities enough for the study.

### Time- and dose–dependent anti-proliferative effects of Nar on human breast and colorectal cancer cells and normal human fibroblast

Cell viability was measured using MTT assay, which is based on the conversion of MTT to formazan crystals by mitochondrial dehydrogenases [14]. Briefly, human colorectal (SW1116, SW837) and breast (HTB26, HTB132) cancer cell lines and normal human fibroblast cells (CRL1554) were seeded (27 × 10^3^ cells/well) in 96-well plates and incubated in a non-CO_2_ or CO_2_ incubator for 18 h, depending on the cancer cell type and medium used. The cells were then treated with various concentrations of Nar (0.05 - 4 mM) for 6–24 h. Untreated cells, to which only DMSO was added at a final concentration of 0.2%, were used as a control. MTT solution (5 mg/ml, filtered) was added to the incubated cells (20 μl/well); the cells were then incubated for another 4 h, and the medium was discarded. DMSO (200 μl) was added to each well, and absorbance was measured in a micro-plate reader at a λ492 nm. The percentages of cell viability and cytotoxicity were calculated using the following equations: % cell viability = (OD_492_ of treated sample/OD_492_ of untreated sample) × 100; % cytotoxicity = 100 - (OD_492_ of treated sample/OD_492_ of untreated sample) × 100).

### Colony forming assay

The antiproliferative effects of Nar on human colorectal (SW1116 and SW837) and breast (HTB26 and HTB132) cancer cell lines were confirmed using a colony-formation assay. Briefly, cancer cells (5 × 10^5^/well) were plated in a 24-well plate and incubated at 37°C in a non-CO_2_ incubator for 24 h. Thereafter, the cells were treated with Nar (3 mM) and incubated at 37°C for 24 h. After that, untreated and treated human cancer cells were collected by trypsinization and washed with Hank's Balanced Salt Solution (HBSS), counted, and plated in 24-well plates at 500 cells/well and incubated at 37°C for 10–14 days. The colonies were washed with cold phosphate-buffered saline (PBS), fixed with 100% methanol, and stained with 0.5% crystal violet. The colonies were counted using an inverted microscope. The data are presented as the number of colonies formed with and without treatment.

### Cell cycle analysis by flow cytometry

The distribution of cell-cycle phases (G_0_/G_1_, S, and G_2_/M) was determined using flow cytometry by measuring the DNA content of nuclei labeled with propidium iodide as described previously [15]. Briefly, human colorectal (SW1116 and SW837) and breast (HTB 26 and HTB132) cancer cell lines were plated (5 × 10^5^ cells/ml) in 24-well plates and incubated at 37°C in a non-CO_2_ incubator for 18 h. The cells were then treated with Nar (3 mM) for 24 h. Untreated and treated human cancer cells were collected by trypsinization, washed with cold phosphate-buffered saline (PBS) and counted. Cells were processed using a DNA-prep kit (Beckman & Coulter) and a DNA-Prep EPICS workstation (Beckman & Coulter). During this process, the cells were treated with a cell-membrane permeabilizing agent (non-ionic detergent) followed by propidium iodide (PI) and RNase. The samples were incubated at room temperature for 15 min before analysis by flow cytometry (FC500, Beckman & Coulter). The percentages of cells in different cell cycle phases were calculated using the Phoenix statistical software package, advanced DNA cell-cycle software (Phoenix Flow System, San Diego, CA).

### DNA fragmentation assay

Induction of apoptosis was monitored using a DNA fragmentation assay [16]. Briefly, human colorectal (SW1116 and SW837) and breast (HTB 26 and HTB 132) cancer cell lines were plated (5 × 10^5^ cells/ml) into 24-well plates and incubated at 37°C in non-CO_2_ incubator for 18 h. Cells were treated with Nar (3 mM) and then collected by trypsinization, washed with cold phosphate-buffered saline (PBS) and centrifuged (1000 × g, 5 min). The cells were then resuspended in 200 μl of DNA lysis buffer (10 mM EDTA, 0.5% Triton X-100, 0.5 mg/ml proteinase K and 50 mM Tris–HCl, pH 8). The lysate was immediately incubated for 24 h at 56°C. After adding phenol/chloroform/isoamyl alcohol (25:24:1) to the lysate, DNA was precipitated with 100% ethanol. The suspension was centrifuged, and the isolated DNA was dissolved in 100 μl of TE buffer. The DNA samples were then loaded onto a 1% agarose gel containing 0.1% ethidium bromide and electrophoresed for 2 h at 70 V. The DNA bands were visualized under UV illumination.

### Annexin V/PI double staining assay for apoptosis

Induction of apoptosis was determined by Annexin V-FITC apoptosis detection kit (BD Hoffmann-La Roche Inc.) according to the manufacturer's instructions. Briefly, human colorectal (SW1116 and SW837) and breast (HTB26 and HTB132) cancer cell lines were plated (5 × 10^5^ cells/ml) in 24-well plate and incubated at 37°C in a non-CO_2_ incubator for 18 h. The cells were treated with Nar (3 mM) for 48 h. Cells from control and treatment groups were re-suspended in 100 μl of staining solution containing annexin V fluorescein and propidium iodide in HEPES buffer (Annex V-FITC, BD Pharmingen, San Diego, CA). Following incubation at room temperature for 15 min, cells were analyzed by flow cytometry. Annexin V binds to those cells that express phosphatidylserine (PS) on the outer layer of the cell membrane, and propidium iodide stains the cellular DNA of those cells with a compromised cell membrane. This technique allows for the discrimination of live cells (unstained with either fluorochrome) from apoptotic cells (stained only with annexin V) and necrotic cells (stained with both annexin V and propidium iodide).

### Western blot analysis

Expression of PI3K, phospho-Akt1/2/3 (Ser473), Akt, NFκBp65, pIκBα and β-actin at the level of translation was determined by western blot analysis as recently described [15] with some modifications. Human colorectal (SW1116) and breast (HTB26) cancer cell lines were plated (5 × 10^5^ cells/ml) in 24-well plates and incubated at 37°C in a non-CO_2_ incubator for 18. The cells were treated with Nar (3 mM) for 24 h. Untreated and Nar-treated cancer cells were scraped from the wells and collected by centrifugation (1000 × g, 5 min, 4°C). The cells were then washed with ice-cold phosphate-buffered saline (PBS) and re-centrifuged. Nuclear fraction (NFκB) was prepared using cytosol/nuclear fractionation kit (BioVision, Inc.) according to the manufacturer instructions. Whole cell extract (PI3K, phospho-Akt, Akt, pIκBα and β-actin) was prepared as recently described (15). Briefly, the cell pellets obtained were re-suspended and lysed in buffer containing 20 mM Tris (pH 7.4), 200 mM sodium chloride, 1 mM ethylenediaminetetraacetic acid (EDTA), 10 mM β-glycerophosphate, 10 mM sodium pyrophosphate, 0.5% NP-40, 0.05% sodium deoxycholic acid, and protease inhibitor cocktail, and the lysates were clarified by centrifugation at 17000 × g for 30 min at 4°C. Protein concentrations of nuclear fractions and whole cell extracts were measured using the Bradford assay (Bio-Rad, Hercules, CA). Untreated and Nar-treated nuclear or whole cell extracts were mixed with equal volumes of 5 × SDS sample buffer, boiled for 5 min, and separated by 10-12% sodium dodecyl sulfate-polyacrylamide gel electrophoresis (SDS-PAGE). After electrophoresis, proteins were transferred to polyvinylidene difluoride (PVDF) membranes (Millipore, Billerica, MA). The membranes were blocked in 5% nonfat dry milk dissolved in PBST (3.2 mM Na_2_HPO_4_, 0.5 mM KH_2_PO_4_, 1.3 mM KCl, 135 mM NaCl, 0.05% Tween-20) overnight at 4°C. Primary antibodies specific to PI3K, phosph-Akt1/2/3 (Ser473), Akt, pIκBα and β-actin (Santa Cruz, CA) were adsorbed to the membranes of whole cell extracts and primary antibody specific to NFκBp65 was adsorbed to the membrane of nuclear fractions. After washing with PBS containing 0.1% Tween 20, the blots were incubated with an alkaline phosphatase-conjugated species-specific IgG secondary antibody for 2 h at room temperature. Bound antibodies were detected using nitroblue tetrazolium and bromochloroindoyl-phosphate. The specificities of the antibodies used in this study were examined by testing their reactivity with unrelated antigens, such as bovine serum albumin (BSA). The signal intensities of the respective bands were quantified by GS-800 calibrated imaging densitometer (Bio-Rad Laboratories, Inc. CA, USA).

### Relative quantitative Real-Time PCR assay

mRNA expression of cell cycle- and apoptosis-associated genes in control and Nar-treated cells was determined by real-time polymerase chain reaction (qRT-PCR) using an ABI 7000 SDS system (Applied Biosystems, USA) and the comparative ∆∆Ct method [15]. Commercial assays that target specific genes with probes and primers were obtained from Applied Biosystems. The targets and their Applied Biosystems assay numbers for the cell-cycle regulatory genes were as follows: *cdk4* (assay ID: Hs00364847_m1); *cdk6* (assay ID: Hs00608037_m1.); *Cdk7* (assay ID: Hs00361486_m1); *p18* (assay ID: Hs00176227_m1); *p19* (assay ID: Hs00176481_m1); and *p21* (assay ID: Hs00355782_m1). The targets and their Applied Biosystems assay numbers for the pro-apoptotic, anti-apoptotic and caspase genes were as follows: *Bax* (assay ID: Hs00180269_m1); *Bak* (assay ID: Hs00832876_m1); *AIF* (assay ID: Hs00269879-m1); *c-IAP-2* (assay ID: Hs00985029_m1); *Bcl2* (assay ID: Hs00608023_m1); *×−IAP* (Assay ID: Hs00236913_m1); *casp3* (assay ID: Hs00234387_m1); *casp7* (assay ID: Hs00169152_m1); *casp8* (assay ID: Hs01018151-m1); and *casp9* (assay ID: Hs00154260_m1). *GAPDH* (assay ID: Hs99999905_m1) was used as an endogenous control to normalize the expression values for each sample. For the comparative Ct method, we performed a two-step RT-PCR to obtain cDNA and real-time quantification using the target gene expression assays and Taqman Universal Master Mix (Applied Biosystems). Colorectal and breast cancer cells (5 × 10^5^ cells/ml) were plated in 24-well plates and incubated in a non-CO_2_ incubator for 18 h. The cells were then treated with Nar (3 mM) for 24 h. mRNA was extracted using the nucleospin RNAII ready-to-use system (MACHEREY-NAGEL), and 200 ng/μl of mRNA was used in the RT reaction. Contaminated DNA was eliminated with DNase-I treatment for 20 min at 25°C, followed by heat inactivation for 10 min at 65°C, prior to cDNA synthesis using the high-capacity cDNA reverse transcription kit (Applied Biosystems) according to the manufacturer's instructions. For each sample, 2.5 μl of cDNA and 12.5 μl of Taqman Universal Master Mix (2×) were used, and the final volume was adjusted to 25 μl with nuclease-free water on an optical 96-well reaction plate (Applied Biosystems). Real-time PCR was performed on an ABI 7000 SDS system using ABI Prism's SDS collection software version 1.1 (Applied Biosystems). Real-time PCR conditions followed the protocol given by the manufacturer of the Taqman Universal Master Mix: step 1, 95°C for 10 min; step 2, 94°C for 15 s; and step 3, 60°C for 1 min. The samples were analyzed using SDS collection software version 1.1, with the baseline set between 3 and 15 and the threshold set at 0.2. The amount of target normalized to an endogenous reference and relative to a calibrator (untreated) was determined by 2^-∆∆Ct^, and the log comparative Ct is presented graphically.

### Nar enhances the anticancer effects of DNA-acting drugs on colorectal and breast cancer cells

The potential of Nar to sensitize human colorectal (SW1116, SW837) and breast (HTB26, HTB132) cancer cells to DNA-acting drugs was studied as previously described [17]. Briefly, cancer cells were plated (27 × 10^3^ cells/well) in 96-well plates at 37°C in a non-CO_2_ incubator. Eighteen hours after culture initiation, the cells were simultaneously treated for 24 h with Nar (1.0 mM) and various concentrations of the following DNA-damaging drugs: camptothecin (CPT), 5-fluorouracil (5FU), doxorubicin (DOX), cis-platin (CIP), ellipticine (ELP), and etoposide (ETP) at concentrations of 1 × 10^−10^ - 1 × 10^−3^ M; carboplatin (CAP) at concentrations of 1 × 10^−10^ - 1 × 10^−4^ M; and cyclophosphamide (CPA) at concentrations of 1× 10^−11^ - 1 × 10^−5^ M. The drugs were then removed, the cells were washed with HBSS, and cell growth was monitored using an MTT assay.

### Statistical analyses

The results are expressed as the mean ± SEM of at least three independent experiments. Statistical analyses were performed with SPSS-21. The statistical significance of the differences between the control and treated groups were determined by one-way ANOVA. *P* values < 0.05 were considered significant.

## Results

### Isolation and identification of Nar

Nar was isolated as yellow needles (EtOAc-ether): mp 240–242°C; UV(MeOH) λ_max_ (log ε) 240 (3.75) nm; IR (KBr) ν_max_ 3343, 1639, 1562, 1515, 1419 cm^−1^; ^1^H NMR (CD_3_OD, 600 MHz) see Table 1; ^13^C NMR (CD_3_OD, 150 MHz) see Table 1; EIMS (70 eV) *m*/*z* 272 [M]^+^.

**Table 1:** **NMR Data of Nar** ^**a**^

**Position**	**δ**_**H**_ **(m,** ***J*** **Hz)**	**δ**_**C**_ **, m** ^***b***^
2	5.33 (dd, 13.2, 3.0)	80.5, d
3	3_ax_ 3.11 (dd, 13.2, 17.1)	44.0, t
	3_eq_ 2.69 (dd, 17.1, 3.0)	
4	-	197.8, s
5	-	165.5, s
6	5.90 (d, 1.8)	96.2, d
7	-	168.4, s
8	5.89 (d,1.8)	97.1, d
9	-	164.9, s
10	-	103.4, s
1′	-	131.1, s
2′	7.32 (dd, 7.2, 1.8)	129.1, d
3′	6.83 (dd, 6.6, 2.4)	116.3, d
4′	-	159.0, s
5′	6.83 (dd, 7.2, 1.8)	116.3, d
6′	7.32 (dd, 7.2, 1.8)	129.1, d
OH	4.89 (s)	-

^*a*^Spectra recorded in methanol-*d*_4_. ^*b*^ ^13^C multiplicities were determined by DEPT 135°.

### Anti-proliferative effects of Nar on human colorectal and breast cancer cells

**Figure 1:**
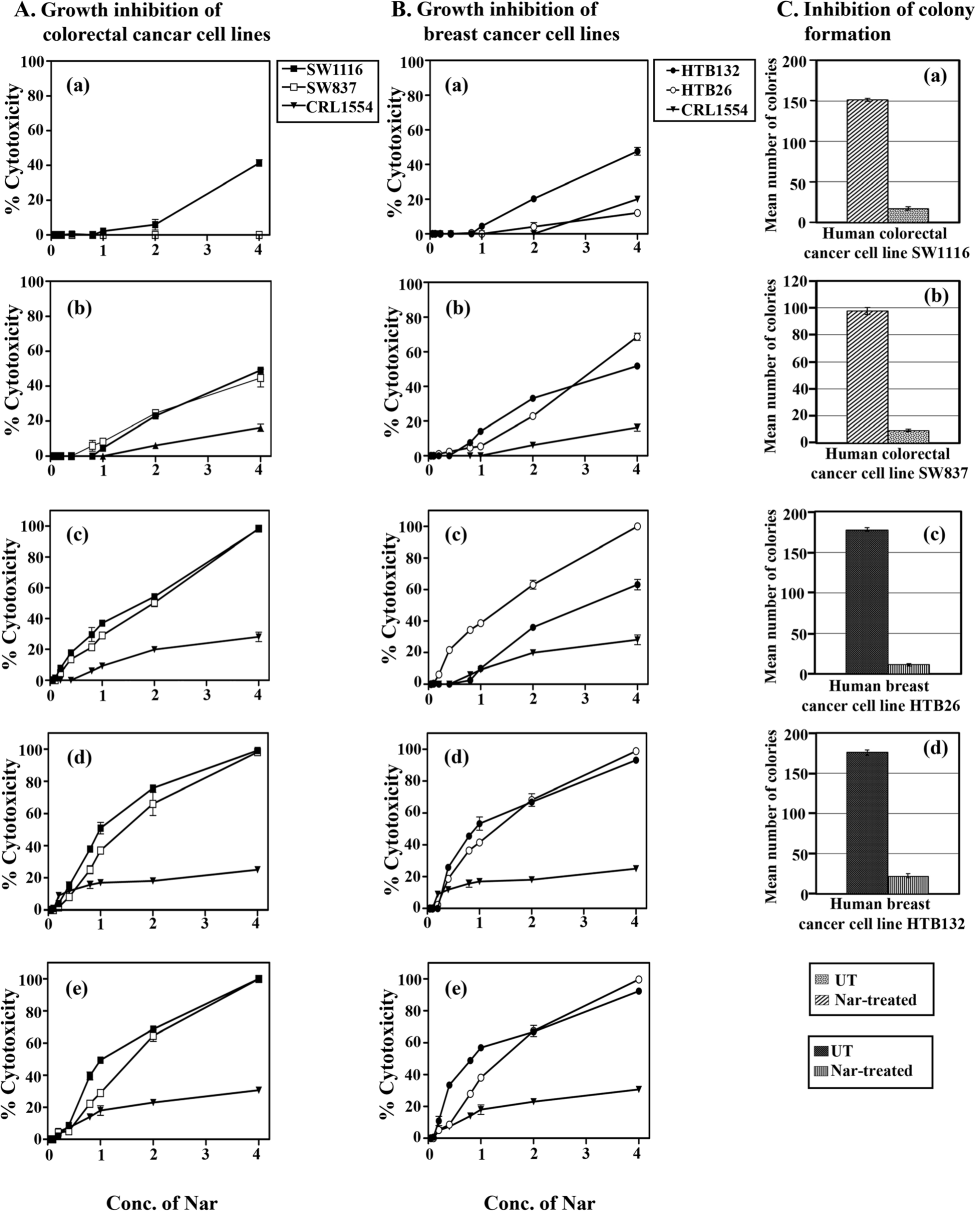
Time and dose-dependent anti-proliferative effects and inhibition of colony formation of Nar on human colorectal and breast cancer cell lines. Human colorectal cancer cells (SW1116, SW837) **(A a-** **e)**, human breast cancer cells (HTB 26, HTB132) **(B a-** **e)** and normal human fibroblast cells (CRL1554) **(A,** **B a-** **e)** were plated (27 × 10^3^ cells/well) in 96-well plates in CO_2_ and non-CO_2_ incubators, depending on type of media and cells, at 37°C for 18 h. The cells were then treated with various concentrations of Nar (0.05 – 4.0 mM) for 3–24 h. Cell growth was monitored using an MTT assay. Untreated and Nar-treated colorectal cancer cells SW1116 **(Ca)**, SW837 **(Cb)** and Nar-treated breast cancer cells HTB26 **(Cc)**, HTB132 **(Cd)** were trypsinized, counted and plated (500 cells /well) in 6-well plates. Following 10–14 days of incubation in non-CO_2_ incubator at 37°C, the colonies were fixed and stained with crystal violet. The stained colonies were counted and compared with the untreated control.

To investigate the effect of Nar on human colorectal and breast cancer cells, cell viability was studied using an MTT assay. Treatment of human colorectal (SW1116, SW837) and breast (HTB26, HTB132) cancer cell lines with various concentrations of Nar (0.5-4 mM) for 6–24 h showed time- and dose-dependent anti-proliferative effects (Figure 1A, B). Treatment of SW1116 with Nar for 6 and 12 h produced 42 ± 2.8% (Figure 1 Aa) and 52 ± 3.5% (IC_50_ = 4 mM) (Figure 1Ab) growth inhibition, respectively. Nar did not show any effect on normal human fibroblast cells (CRL1554) at 6 and 12 h, and the difference between the effects of Nar on SW1116 and CRL1554 was significant at 6 h (*P* ≤ 0.008) and non-significant at 12 h (*P* ≤ 0.093). In addition, treatment of SW1116 cells with Nar for 15 h markedly inhibited their growth (% cytotoxicity = 0.0 - 98%, *P* ≤ 0.004, IC_50_ = 1.73 mM) compared with its effect on CRL1554 cells (% cytotoxicity = 0.0 - 34%) (Figure 1Ac). Moreover, exposure of SW1116 cells to Nar for 18 h showed significantly greater (% cytotoxicity = 0.0 -99.4%, *P* ≤ 0.007, IC_50_ = 0.91 mM) growth inhibition than that exerted on CRL1554 cells (% cytotoxicity = 0.0 - 26%) (Figure 1Ad). Furthermore, treatment of SW1116 cells with Nar for 24 h produced a higher growth inhibition (% cytotoxicity = 0.0- 100%, IC_50_ = 1.0 mM) than that produced on CRL1554 cells (0.0 - 32%). The difference in the growth inhibition was significant (*P* ≤ 0.015) (Figure 1Ae).

Treatment of SW837 with various concentrations of Nar for 12 h produced a higher growth inhibition (% cytotoxicity = 0.0 - 55%, *P* ≤ 0.06, IC_50_ = 4.0 mM) than that produced on CRL1554 cells (% cytotoxicity = 0 - 19%) (Figure 1Ab). In addition, treatment of SW837 cells with Nar for 15 h produced a higher cytotoxicity (% cytotoxicity = 0.0 -99.5%, *P* ≤ 0.015, IC_50_ = 2.0 mM) than that exerted on CRL1554 cells (% cytotoxicity = 0- 34%) (Figure 1Ac). Moreover, exposure of SW837 cells to Nar for 18 h markedly inhibited their proliferation (% cytotoxicity = 0.0 – 99.9%, *P* ≤ 0.042, IC_50_ = 1.36 mM) compared with its effect on CRL1554 cells (% cytotoxicity = 0.0 - 26%) (Figure 1Ad). Further incubation of SW837 cells with Nar for 24 h produced a higher growth inhibition (% cytotoxicity = 0.0 - 100%, *P* ≤ 0.066, IC_50_ = 1.55 mM) than that produced on CRL1554 cells (% cytotoxicity = 0.0 - 32%) (Figure 1Ae).

Treatment of the human breast cancer cell line HTB26 with Nar (0.2-4 mM) for 6 h exhibited a slight growth inhibition (% cytotoxicity = 0 - 13%, *P* ≤ 0.897) with no effect on CRL1554 cells (Figure 1Ba). A higher growth inhibitory effect was observed on HTB132 cells (% cytotoxicity = 0.0 - 52%, *P* ≤ 0.035, IC_50_ = 4.0 mM) (Figure 1Ba.) after exposure to Nar for 6 h. Exposure of HTB26 cells for 12 h produced a substantially higher growth inhibition (% cytotoxicity = 0.0 – 73%, *P* ≤ 0.041, IC_50_ = 3.09 mM) compared with CRL1554 cells (% cytotoxicity = 0.0 – 19%). A similar growth inhibition was noted with HTB132 cells treated with Nar for 12 h (% cytotoxicity = 0.0 – 55%, *P* ≤ 0.038; IC_50_ = 3.73 mM) (Figure 1Bb). Moreover, exposure of HTB26 to Nar for 15 h markedly inhibited its growth (% cytotoxicity = 0.0 – 100%, P ≤ 0.001, IC_50_ = 1.36 mM) compared with CRL1554 cells (% cytotoxicity = 0.0 – 34%). Exposure of HTB132 cells to Nar for 15 h produced a higher anti-proliferative effect (% cytotoxicity = 0.0 – 67%, *P* ≤ 0.007, IC_50_ = 3.0 mM) than that produced on CRL1554 cells (Figure 1Bc). In addition, exposure of HTB26 (% cytotoxicity = 0.0 – 99.6%, *P* ≤ 0.011, IC_50_ = 1.46 mM) and HTB132 (% cytotoxicity = 0.0 – 95%, *P* ≤ 0.005, IC_50_ = 0.91 mM) cells to Nar for 18 h produced greater growth inhibition than that observed in CRL1554 cells (% cytotoxicity = 0.0 – 26%) (Figure 1Bd). Furthermore, a similar significant growth inhibition was observed in HTB26 (% cytotoxicity = 0.0 – 99.9%, *P* ≤ 0.024, IC_50_ = 0.82 mM) and HTB132 (% cytotoxicity = 0.0 – 95.3%, *P* ≤ 0.002, IC_50_ = 0.36) cells compared with CRL1554 cells (% cytotoxicity 0.0 – 32%) after a 24-h exposure to Nar (Figure 1Be).

The anti-proliferative effects of Nar on human colorectal (SW1116, SW837) and breast (HTB26, HTB132) cancer cells were confirmed by the inhibition of colony formation. Treatment of SW1116 cells with Nar markedly inhibited colony formation (mean number of colonies = 17 ± 2, *P* ≤ 0.0001) compared with untreated SW1116 cells (mean number of colonies = 152 ± 1.84) (Figure 1Ca). Additionally, exposure of SW837 cells to Nar greatly inhibited colony formation (mean number of colonies = 9 ± 0.9, *P* ≤ 0.0001) compared with untreated SW837 cells (mean number of colonies = 98 ± 2.6) (Figure 1Cb).

Exposure of HTB26 cells to Nar showed a marked inhibition of colony formation (mean number of colonies = 11 ± 1.6, *P* ≤ 0.0001) compared with untreated HTB26 cells (mean number of colonies = 177 ± 3) (Figure 1Cc). Similar marked inhibition of colony formation was observed with Nar-treated HTB132 cells (mean number of colonies = 22 ± 1.8, *P* ≤ 0.0001) compared with untreated HTB132 cells (mean number of colonies = 176 ± 3) (Figure 1Cd).

### Nar arrests the growth of human colorectal and breast cancer cells

**Figure 2:**
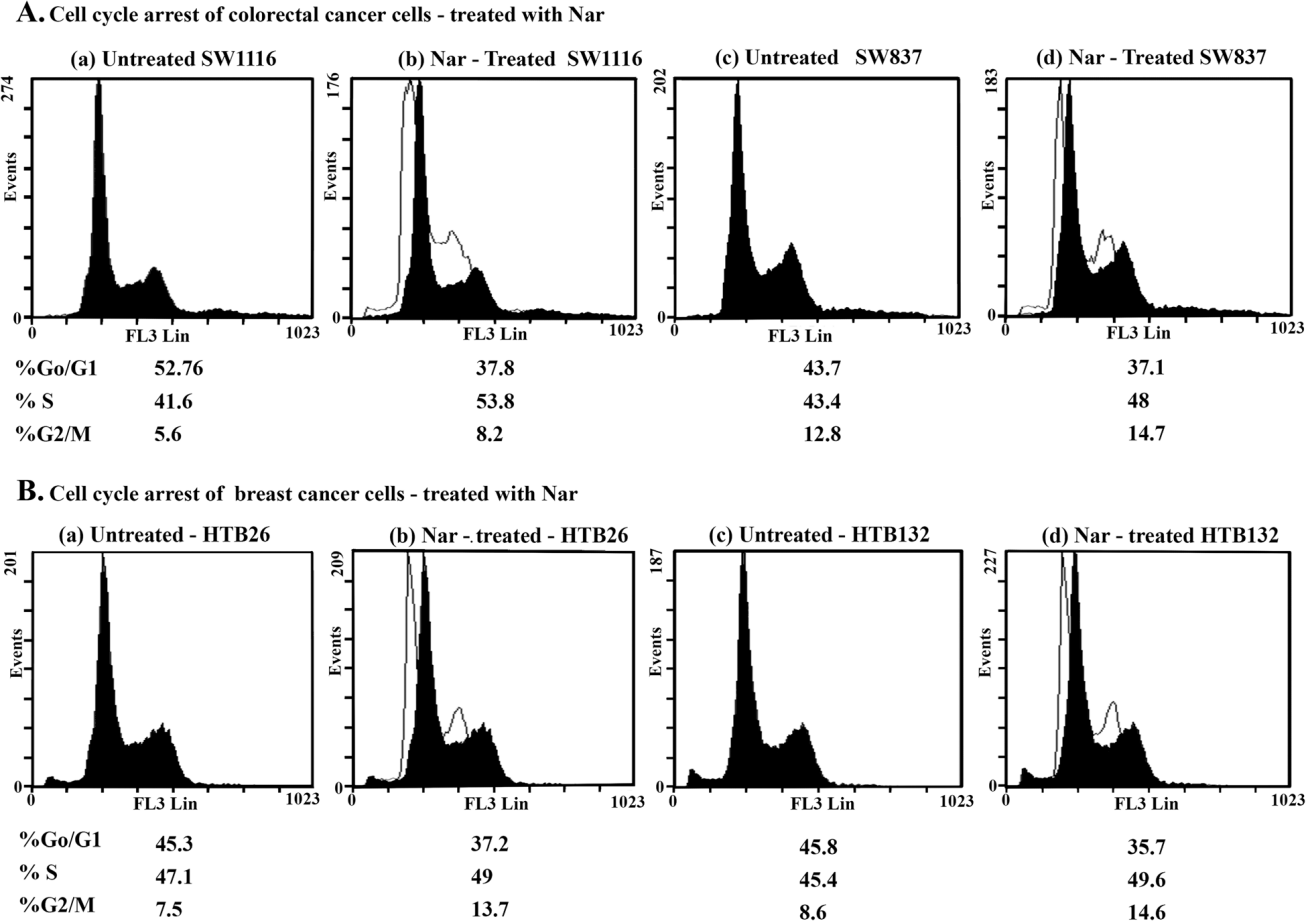
Analysis of cell cycle perturbation of human colorectal and breast cell lines treated with Nar. Human colorectal cancer cells **(A)** and human breast cancer cells **(B)** were plated (2.5 × 10^5^ cells/well) into 24-well plates in a non-CO2 incubator at 37°C for 18 h. The cells were then treated with Nar (3.0 mM) for 24 h. At least three samples were analyzed, and 20,000 events were scored for each sample. The vertical axis represents the relative number of events, and the horizontal axis represents the fluorescence intensity.

To further demonstrate that the growth inhibition of human colorectal and breast cancer cells leads to alterations of the cell cycle distribution, cells were treated with Nar and stained with PI. The percentage of cells in each stage of the cell cycle was analyzed using flow cytometry. Treatment of the human colorectal cancer cell line SW1116 with Nar for 24 h resulted in accumulation of cells in S-phase (53.8% ± 2.2 vs. 41.6% ±1.8 for untreated; UT, *P* ≤ 0.005) and G_2_/M-phase (8.2% ± 1.3 vs. 5.6% ± 1.3 for UT, *P* ≤ 0.204) with a corresponding decrease in the fraction of cells in G_0_/G_1_ phase (37.8% ± 1.7 vs. 52.76% ± 1.2 for UT, *P* ≤ 0.001) (Figure 2Aa, b). Furthermore, Nar growth-arrested SW837 cells in both S-phase (48% ± 1.73 vs. 43.4% ± 1.96 for UT, *P* ≤ 0.123) and G_2_/M-phase (14.7% ± 1.2 vs. 12.8% ± 1.1 for UT, *P ≤* 0.471) with a corresponding decrease of cells in G_0_/G_1_-phase (37.1% ± 1.2 vs. 43.7% ± 2.7 for UT, *P* ≤ 0.045) (Figure 2Ac, d).

On the other hand, treatment of the human breast cancer cell line HTB26 with Nar resulted in accumulation of HTB26 cells in both S-phase (49% ± 1.2 vs. 47.1 ± 1.2 for UT, *P* ≤ 0.289) and G_2_/M-phase (13.7% ± 1.2 vs. 7.5% ± 1.4 for UT, *P* ≤ 0.013), with a corresponding decrease of cells in G_0_/G_1_-phase (37.2% ± 1.7 vs. 45.3% ± 1.9 for UT, *P* ≤ 0.019) (Figure 2Ba, b). Additionally, exposure of HTB132 cells to Nar resulted in accumulation of HTB132 cells in S-phase (49.6% ± 2.3 vs. 45.4% ± 2.3 for UT, *P* ≤ 0.246) and G_2_/M-phase (14.6% ± 1.7 vs. 8.6% ± 1.2 for UT, *P* ≤ 0.035) with a corresponding decrease of cells in G_0_/G_1_-phase (35.7% ± 2.3 vs. 45.8% ± 2.3 for UT, *P* ≤ 0.021) (Figure 2Bc, d).

### Nar induces apoptosis in human colorectal and breast cancer cells

Agents with the ability to induce apoptosis in tumors have the potential to be used for antitumor therapy. The effect of Nar (3 mM) on the induction of programmed cell death in human breast (HTB26 and HTB132) and colorectal (SW1116 and SW837) cancer cell lines was assessed with DNA fragmentation analysis. The results from DNA gel electrophoresis showed the formation of DNA laddering in both breast and colorectal cancer cell lines treated with Nar (data not shown).

To investigate the type of cell death induced by Nar, cells were stained with annexin V-FITC/PI and analyzed by flow cytometry. Annexin V is a Ca^2+^-dependent phospholipid-binding protein possessing a high affinity for PS, a membrane-bound component localized to the inner surface of the cell membrane. An indicator of early-stage apoptosis is the detection of exposed PS residues that have translocated to the cell surface. The annexin V assay permits the simultaneous detection of early apoptotic events based on annexin V binding to exposed PS and late apoptotic/dead events through the uptake of propidium iodide.

**Figure 3:**
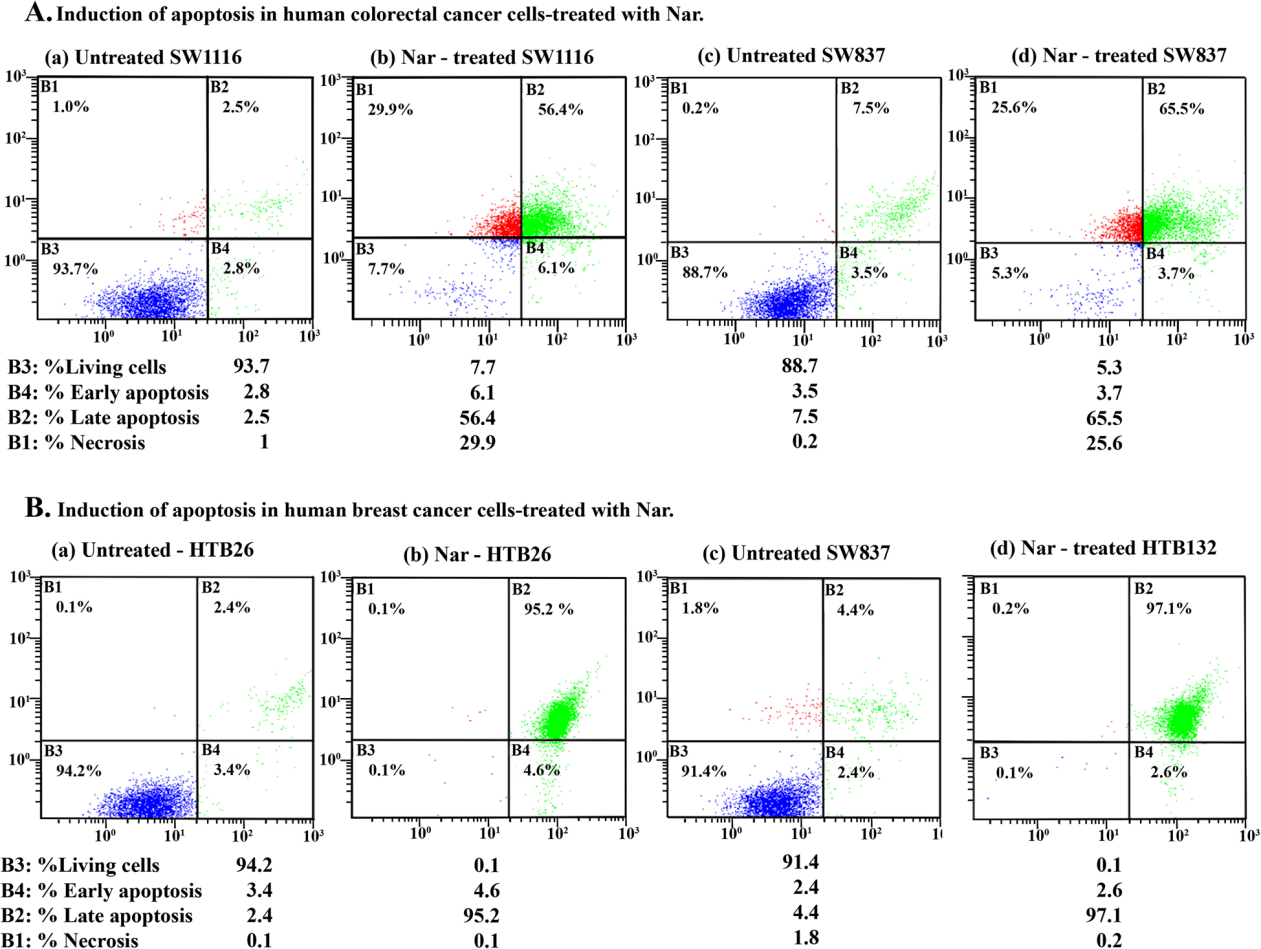
Induction of apoptosis in human colorectal and breast cancer cells treated with Nar. Human colorectal cancer cells **(A)** and human breast cancer cells **(B)** were plated (2.5 × 10^5^ cells/well) into 24-well plates in non-CO2 incubator at 37°C for 18 h. The cells were then treated with Nar (3.0 mM) for 48 h. The cells were processed and stained with annexin V-FITC/PI and analyzed by flow cytometry as described in the Materials and Methods section.

Treatment of SW1116 cells with Nar induced apoptosis, including early apoptosis (6.1% vs. 2.8% for UT), late apoptosis (56.4% vs. 2.5% for UT) and necrosis (29.9% vs. 1% for UT) (Figure 3Aa, b). Similar results were obtained with SW837 cells treated with Nar, which showed early apoptosis (3.7% vs. 3.5% for UT), late apoptosis (65.5% vs. 7.5% for UT) and necrosis (25.6% vs. 0.2% for UT) (Figure 3Ac, d). Moreover, exposure of the human breast cancer cell line HTB26 to Nar induced apoptosis, including early apoptosis (4.6% vs. 3.4% for UT), late apoptosis (95.2% vs. 2.4% for UT) and necrosis (0.1% vs. 0.1% for UT) (Figure 3Ba, b). Similar results were obtained with the breast cancer cell line HTB132, in which Nar induced apoptosis with early apoptosis (2.6% vs. 2.4% for UT), late apoptosis (97.1% vs. 4.4% for UT) and necrosis (0.2% vs. 1.2% for UT) (Figure 3Bc, d).

### Nar affects the expression of Akt, pAkt, PI3K, pIκBα and NFκBp65 in colorectal and breast cancer cells

**Figure 4:**
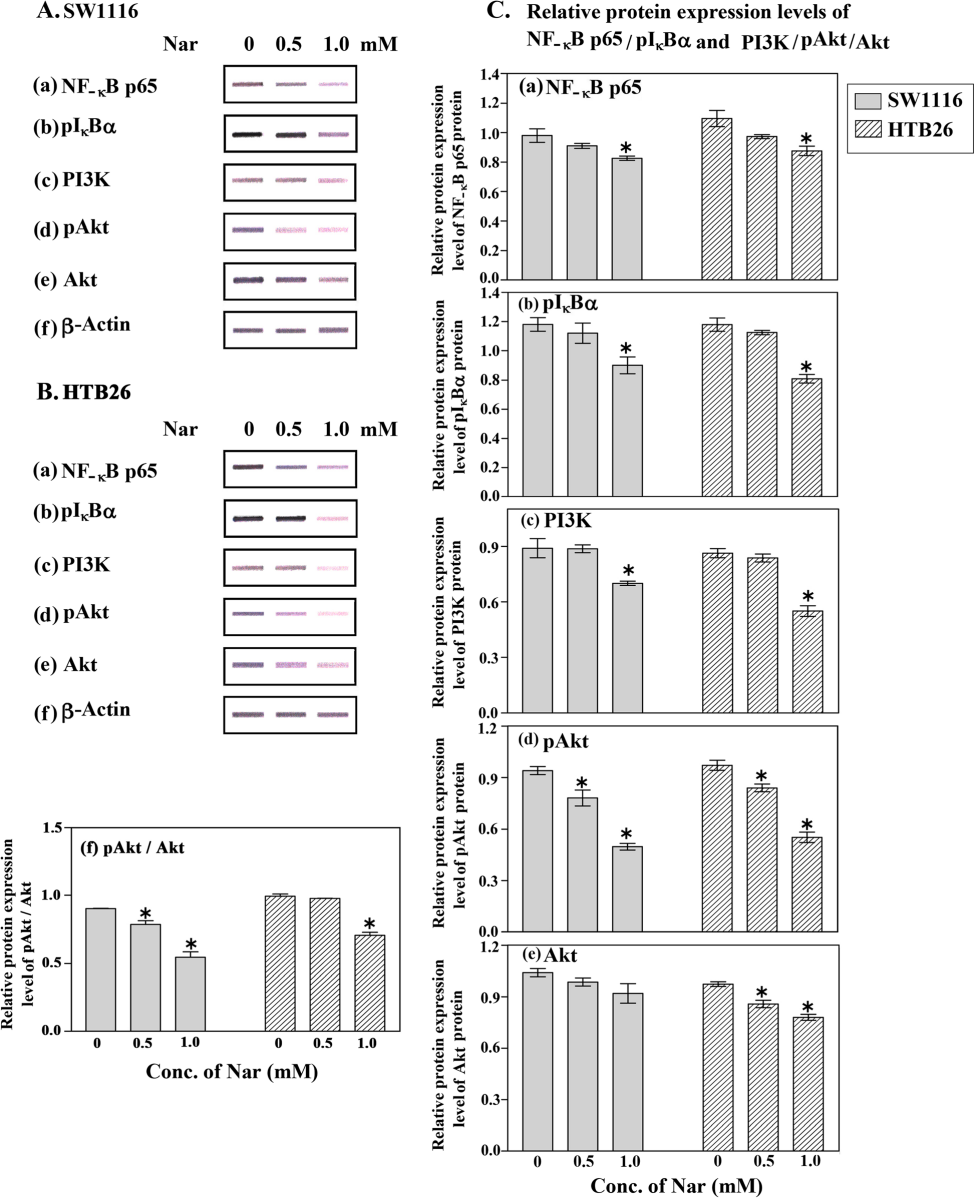
Expression of NKκB, pIκB, PI3K, Akt and pAkt in human colorectal and breast cancer cells treated with Nar. Colorectal (SW1116) and breast (HTB26) cancer cells (2.5 × 10^5^ cells/well) were plated into 24-well plates and incubated at 37°C for 18 h. The cells were then treated with Nar (3 mM) for 24 h. Expression levels of NFκB/pIκB, in the nuclear extracts, **(Aa,** **b** and **Ba,** **b)**; PI3K/pAkt/Akt **(Ac**-**e** and **Bc-** **e)** and β-actin **(Af,** **Bf)**, in the whole cell extracts, were detected by western blot analyses. The relative protein expression of NFκB/pIκB **(C a,** **b)** and PI3K, pAkt, Akt, pAkt/Akt **(Cc-** **f)** was normalized to β-actin. *P ≤ 0.05 vs. control (untreated). The data were obtained from at least duplicate experiments.

Nuclear factor -κB (NF-κB), a major cell survival signal, participates in multiple steps in cancer cell resistance to chemical and radiation therapies. Studies with animal models and cell culture systems have established links between NF-κB and carcinogenesis, highlighting its significance as a target in cancer treatment and chemoprevention [18]. We therefore tested the possibility that Nar might inhibit NF-κB in colorectal and breast cancer cells. Indeed, Nar inhibited the phosphorylation of IκB, an upstream mediator of NF-κB function, and thus, p65 localization into nuclear was also inhibited in colorectal and breast cancer cells treated with Nar (Figure 4a, b), supporting the involvement of NF-κB inactivation in Nar-induced apoptosis. It is known that PI3K and its downstream substrate Akt also have a role in apoptosis [19]. We examined the expression of these proteins, identifying dose-dependent reductions in PI3K (Figure 4c), phospho-Akt (Figure 4d), Akt (Figure 4e) and the ratio of pAkt/Akt in both colorectal and breast cancer cell lines.

### Nar modulates the expression of genes-related to cell cycle and apoptosis in human colorectal and breast cancer cells

Inhibition of the cell cycle and the induction of apoptosis in cancer cells are two major goals in cancer treatment. Therefore, we analyzed the expression of genes controlling both cell cycle and apoptosis in human cancer cells treated with Nar to elucidate the potential molecular anticancer mechanisms of Nar, which remains unclear.

Cell cycle progression in eukaryotic cells is partly controlled by the cyclin-dependent kinase (Cdk) family of protein kinases, their activating partners, the cyclins, and the cyclin-dependent kinase inhibitors (CdkIs) [20].

**Figure 5:**
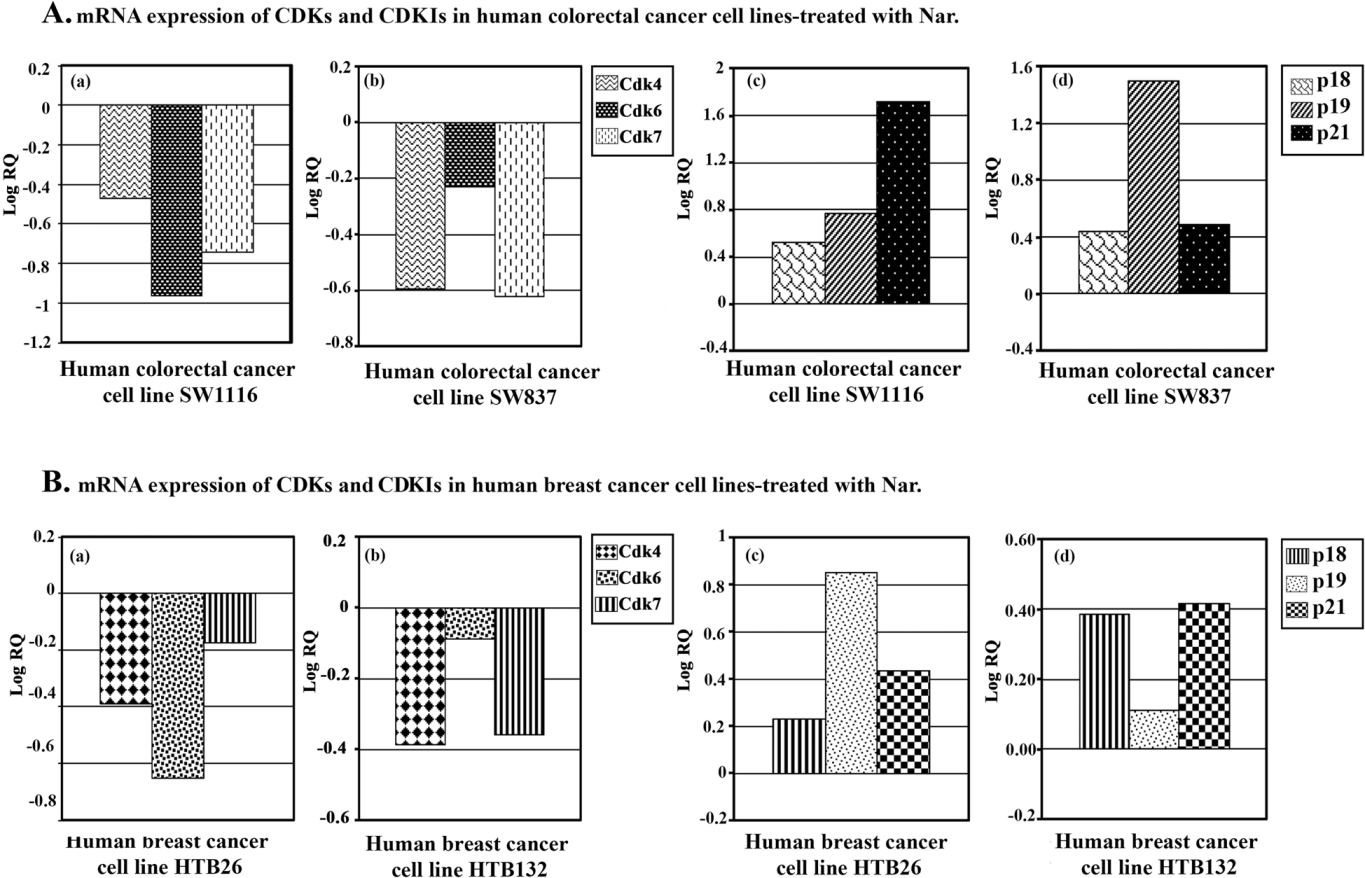
mRNA expression of cyclin-dependent kinase and cyclin-dependent kinase inhibitor genes in human colorectal and breast cancer cells treated with Nar. The expression of cyclin-dependent kinase genes and cyclin-dependent kinase inhibitor genes in human colorectal **(A)** and breast cancer cell lines **(B)** was determined by measuring mRNA levels through real-time RT-PCR and using the comparative ΔΔCt method to calculate expression changes. The amount of the target, normalized to an endogenous reference and relative to calibrator, is given by 2 ^-ΔΔCt^.

Our data showed that Nar differentially downregulated the expression of *Cdk4, Cdk6*, and *Cdk7* in the human colorectal cancer cell lines SW1116 (Figure 5Aa) and SW837 (Figure 5Ab) and in the human breast cancer cell lines HTB26 (Figure 5Ba) and HTB132 (Figure 5Bb). In contrast, Nar differentially upregulated the expression of *p18*, *p19*, and *p21* in both human colorectal cancer cell lines, SW11116 (Figure 5Ac) and SW837 (Figure 5Ad), and breast cancer cell lines, HTB26 (Figure 5Bc) and HTB132 (Figure 5Bd).

On the basis of these observations, we propose that alterations in the level of various cell cycle regulatory proteins were responsible for the cell cycle arrest observed in Nar-induced death in human colorectal and breast cancer cells. Cell cycle arrest may, at least partially, account for the induction of apoptosis and the cytotoxic effects of Nar in human colorectal and breast cancer cells.

**Figure 6:**
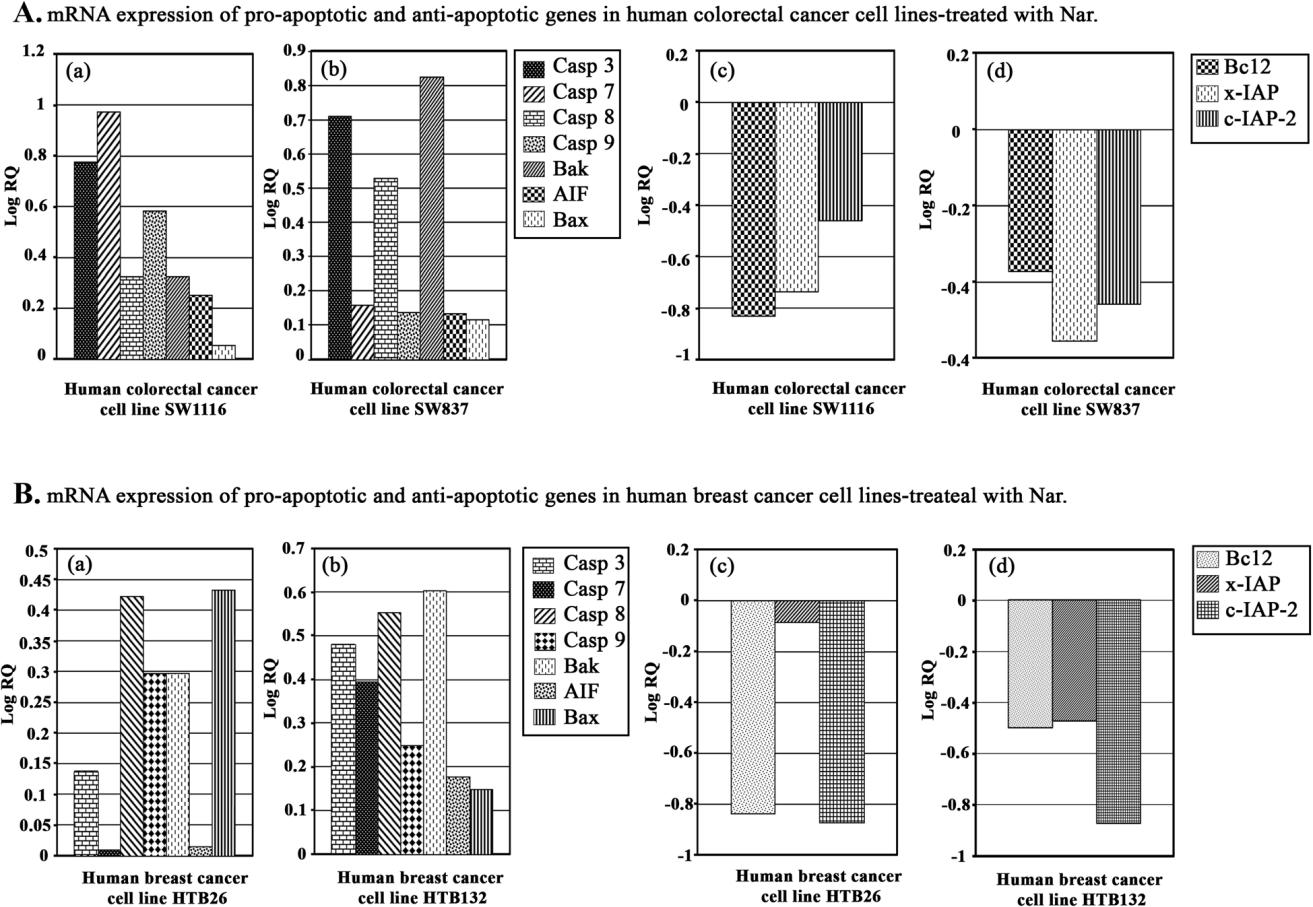
mRNA expression of pro-apoptotic and anti-apoptotic genes in human colorectal and breast cancer cells treated with Nar. The expression of pro-apoptotic and anti-apoptotic genes in human colorectal **(A)** and breast **(B)** cancer cells was determined by measuring mRNA levels through real-time RT-PCR and using the comparative ΔΔCt method to calculate expression changes. The amount of the target normalized to an endogenous reference and relative to calibrator, is given by 2 ^-ΔΔCt^.

Pro-apoptotic and anti-apoptotic proteins are central regulators of apoptosis, and the interactions among these proteins set the threshold for cell survival [21]. Nar differentially up-regulated the expression of the pro-apoptotic genes, including *caspases*-*3*, *7*, *8*, *9*, *Ba*k, *AIF* and *Bax* in SW1116 (Figure 6Aa) and SW837 (Figure 6Ab) as well as HTB26 (Figure 6Ba) and HTB132 (Figure 6Bb). Meanwhile, Nar differentially downregulated anti-apoptotic genes, such as *Bcl2, x-IAP* and *c-IAP-2*, in both SW1116 (Figure 6Ac) and SW837 (Figure 6Ad), as well as HTB26 (Figure 6Bc) and HTB132 (Figure 6Bd). Nar promotes apoptosis in human colorectal and breast cancer cells by altering the ratio of pro and anti-apoptotic genes in favor of apoptosis.

### Nar enhances the sensitivity of colorectal and breast cancer cells to DNA-acting drugs

**Figure 7:**
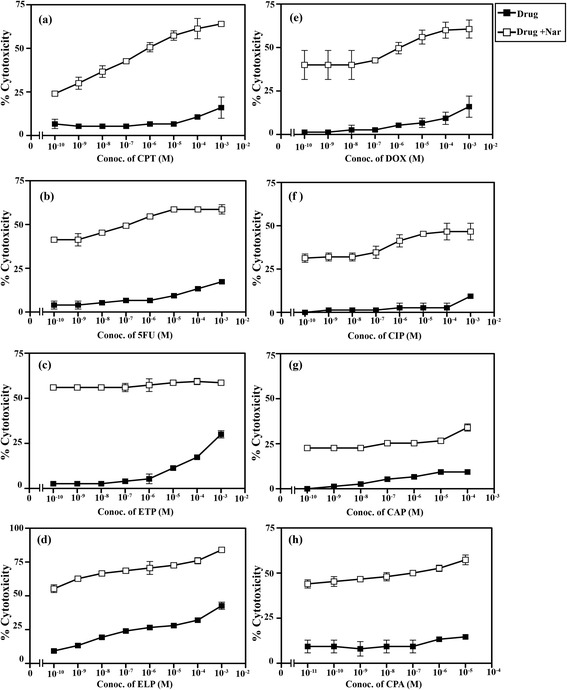
Nar enhances the chemosensitivity of human colorectal cancer cells to DNA-acting drugs. Human colorectal cancer cells SW1116 were plated (27 × 10^3^ cells/well) into a 96-well plate at 37°C in a non-CO_2_ incubator. At 18 h after starting the culture, the cells were treated for 24 h with Nar (1 mM) and various concentrations of camptothecin, CPT doxorubicin, DOX, 5-fluorouracil, 5FU cisplatin, CIP, etoposide, ETP ellipticine, ELP (1 × 10^−10^– 1 × 10^−3^ M), carboplatin, CAP (1 × 10^−10^ – 3.5 × 10^−4^ M) and cyclophosphamide, CPA (1 × 10^−11^ – 1 × 10^−5^ M). Cell proliferation was monitored using an MTT assay.

**Figure 8:**
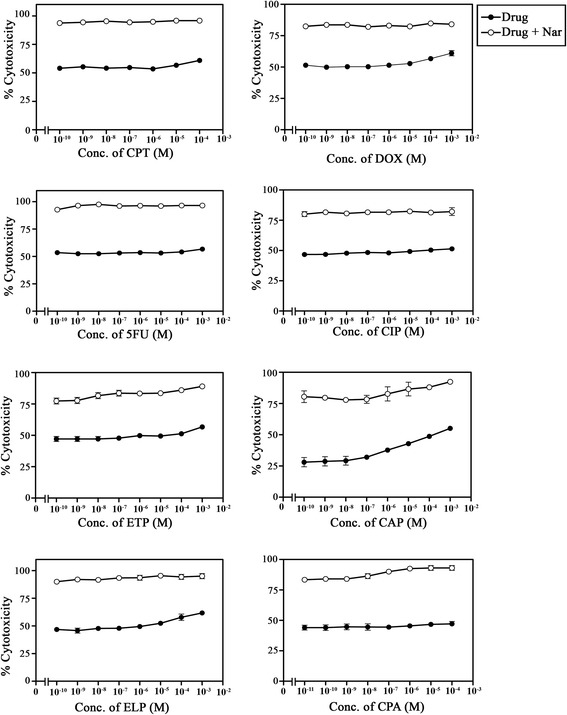
Nar enhances the chemosensitivity of human breast cancer cells to DNA-acting drugs. Human breast cancer cells HTB26 were plated (27 × 10^3^ cells/well) into a 96-well plate at 37°C in a non-CO_2_ incubator. At 18 h after starting the culture, the cells were treated for 24 h with Nar (1 mM) and various concentrations of camptothecin, CPT, doxorubicin, DOX, 5-fluorouracil, 5FU, cisplatin, CIP, etoposide, ETP, ellipticine, ELP (1 × 10^−10^– 1 × 10^−3^ M), carboplatin, CAP (1 × 10^−10^ – 3.5 × 10^−4^ M) and cyclophosphamide, CPA (1 × 10^−11^– 1 × 10^−5^ M). Cell proliferation was monitored using an MTT assay.

The potential of Nar to potentiate the chemo-sensitivity of colorectal and breast cancer cells to DNA-damaging drugs was examined. The results are summarized in Figures 7 and 8 and Table 2. The results reported in this study clearly indicate the potential of Nar to increase the sensitivity of colorectal and breast cancer cells to DNA-damaging drugs. The synergistic and/or additive interaction between the tested DNA-acting drugs and Nar needs to be further investigated and might be dependent on the type of drug tested, the polymorphism of the genes encoding the drug-metabolizing enzymes transporters or drug targets.

**Table 2:** **Percentage mean cytotoxicities of DNA**-**acting drugs and their combinations with Nar on human colorectal and breast cancer cells**

**Treatment with DNA**-**acting drugs and their combinations with Nar**	**Percentage mean cytotoxicity** ^**a**^		**Fold increase in therapeutic efficacy**	
	**SW837**	**HTB132**	**SW837** ^**b**^	**HTB132** ^**c**^
5 FU (1 × 10^−10^ - 1 × 10^−3^ M)	8 ± 1.0	54 ± 0.42		
5 FU (1 × 10^−10^ - 1 × 10^−3^ M) + Nar (0.5 mM)	50 ± 1.6	96 ± 0.44	6.25	1.7
CIP (1 × 10^−10^ - 1 × 10^−3^ M)	3 ± 0.8	49 ± 0.5		
CIP (1 × 10^−10^ - 1 × 10^−3^ M) + Nar (0.5 mM)	39 ± 2	80 ± 0.6	13	1.63
CAP (1 × 10^−10^ - 1 × 10^−3^ M)	9 ± 2	40 ± 2		
CAP (1 × 10^−10^ - 1 × 10^−3^ M) + Nar (0.5 mM)	27 ± 1.0	83 ± 1.5	3	2.1
CPT (1 × 10^−10^ - 1 × 10^−3^ M)	8 ± 1.1	60 ± 2.2		
CPT (1 × 10^−10^ - 1 × 10^−3^ M) + Nar (0.5 mM)	50 ± 2.6	95 ± 0.3	6.25	1.58
DOX (1 × 10^−10^ - 1 × 10^−3^ M)	6 ± 1.3	54 ± 1.3		
DOX (1 × 10^−10^ - 1 × 10^−3^ M) + Nar (0.5 mM)	41 ± 3	84 ± 0.6	6.83	1.56
CPA (1 × 10^−11^ - 1 × 10^−4^ M)	15 ± 2.6	44 ± 0.8		
CPA (1 × 10-11 - 1 × 10-4 M) + Nar (0.5 mM)	47 ± 1.1	88 ± 1	3.13	2
ETP (1 × 10^−10^ - 1 × 10^−3^ M)	10 ± 2	51 ± 1.6		
ETP (1 × 10^−10^ - 1 × 10^−3^ M) + Nar (0.5 mM)	57 ± 0.6	83 ± 0.93	5.7	1.63
ELP (1 × 10^−11^ - 1 × 10^−4^ M)	23 ± 2.5	51 ± 1.6		
ELP (1 × 10^−11^ - 1 × 10^−4^ M) + Nar (0.5 mM)	70 ± 1.8	93 ± 0.6	3.04	1.82

^**a**^The data are based on the mean of absorbance from 3 independent experiments.

^**b**^,^**c**^ *P*-values of the combined treatments with DNA-acting drugs and Nar versus DNA-acting drugs = 0.0001.

## Discussion

The need for new drugs has prompted studies evaluating possible anticancer agents in fruits, vegetables, herbs, and spices. Natural products with diverse bioactivities are becoming an important source of novel agents with pharmaceutical potential. The potential for plant extracts to act as anticancer therapeutic agents is due to their ability to inhibit tumor growth, angiogenesis, and metastasis with few side effects [22]. Much of this activity appears to stem from flavonoids, which are principal components of many such extracts that demonstrate the capacity to inactivate carcinogens, inhibit angiogenesis, and halt cell proliferation or promote apoptosis [23]. Nar, the aglycone of naringin, is a flavonoid with activity against uterine, blood, stomach, brain, and lung cancer cell lines [24] with no toxic effect on normal cells [25,26]. Its inhibitory effects on tumor growth have spurred interest in its therapeutic application. However, the detailed molecular mechanisms of its anti-proliferative effects and apoptosis induction on human colorectal and breast cancer cells remained to be elucidated. In this study, we endeavored to investigate the effects of Nar on human colorectal and breast cancer cell growth, the intracellular transduction pathways that regulate apoptosis and the potential for Nar to enhance the sensitivity of both colorectal and breast cancer cells to DNA-acting drugs.

The dried powdered *Thymus vulgaris* was percolated repeatedly in 96% EtOH at room temperature. The combined extracts were evaporated *in vacuo* until desiccation. A total of 10 g of the dried residue was partitioned successively, between water and ethyl acetate. Flash chromatography of the ethyl acetate fraction over silica gel, using 15% acetone in chloroform as an eluent, yielded the known flavanone Nar [27] in addition to the flavanonol aromadendrin [28], and Nar was isolated as pale yellow needles. Its molecular formula was determined as C_15_H_12_O_5_ on the basis of the ion peak at *m*/*z* 272 [M]^+^ and NMR data. Its IR spectrum showed absorption bands for a hydroxyl group (3343 cm^−1^), a conjugated carbonyl group (1639 cm^−1^) and an aromatic moiety (1562, 1515, and 1419 cm^−1^). The ^1^H and ^13^C NMR spectra identified the possible identity of this compound as a flavanone. The ^13^C NMR spectrum showed fifteen carbon resonances distributed as a triplet, five doublets (two of them are of double intensity) and seven singlets. The double intensity doublets resonated in the aromatic region (at *δ*_C_116.3 and 129.1, Table 1), indicating the presence of a *para*-di-substituted aromatic ring; two resonances of the remaining doublets resonated, again, in the aromatic region of *δ*_C_ 96.2 and 97.1 (Table 1).

The coupling pattern and constants in the ^1^H NMR spectrum confirmed the presence of 1,2,3,5-tetrasubstituted and 1,4-disubstituted aromatic rings (ring A and ring B, respectively). The last doublet resonated in the oxygenated aliphatic region at *δ*_C_ 80.5 ppm (C-2), while the only triplet carbon resonated at *δ*_C_ 44.0 ppm (C-3). This result confirmed the presence of a flavanone ring system. The HSQC spectra showed a cross peak between C-2 (*δ*_C_ 80.5) and a proton resonated at *δ*_H_ 5.33 as a double doublet with *J* = 13.2, 3.0 H_Z_ (H-2). This coupling constant and pattern are only possible when H-2 adapts an axial (*β*-) orientation. Ring B, however, is attached to C-2 with an α-disposition. Therefore, the absolute stereochemistry around C-2 was assigned as *S*. The D_2_O-exchangeable sharp singlet resonating at *δ*_H_ 4.89 indicated the presence of at least one hydroxyl group. Other HSQC, HMBC and COSY spectra facilitated the full unambiguous assignment of the protons and carbons of this compound and revealed its identity as the known flavanone Nar [27].

The anti-proliferative effects of Nar on colorectal (SW1116, SW837) and breast (HTB26, HTB132) cancer cells as well as normal human fibroblast CRL1554 cells were investigated first. Nar was found to be cytotoxic to both colorectal and breast cancer cells in a dose- and time-dependent manner. CRL1554 human fibroblast control cells were slightly affected by Nar. Therefore, Nar appeared to reduce cancer cell growth with minimal collateral damage. These results are in line with those reported in other studies using Nar or Nar derivatives in various cancer cell lines or animal models [29,30]. Our results contradict the recent findings that Nar exhibits no anti-cancer activity against human cervical cancer cells [31]. Very recently, the nano-chemoprevention concept was introduced as a novel approach for improving phytochemical bioavailability and anti-tumor effects. Oral administration of Nar-loaded nanoparticles (NARNPs) to 7,12-dimethylbenz(a)anthracene (DMBA)-treated animals completely prevented tumor formation compared with the free Nar and significantly reduced the degree of histological lesions, in addition to restoration of the status of biochemical and molecular markers during oral carcinogenesis [32]. NARNPs were found to have a more potent antitumor effect than free Nar in completely preventing the formation of squamous cell carcinoma and in restoring the biochemical constituents to normal range [33].

Cell cycle arrest and apoptosis are two important mechanisms involved in anti-cancer drug treatment [34]. Uncontrolled cellular proliferation is a hallmark of all cancer, and the blockade of the cell cycle is regarded as an effective strategy for eliminating cancer cells [35]. Many chemotherapeutic agents have been shown to impart anti-proliferative effects *via* arresting cell division at certain checkpoints in the cell cycle [36]. The concept of cell cycle-mediated apoptosis has gained increasing attention, as targeting this pathway may provide an opportunity to overcome acquired drug resistance, decrease mutagenesis and reduce toxicity [36].

In the present study, cell cycle arrest was observed when cells were cultured with Nar. Characterization of this effect demonstrated that expression levels of cell cycle regulators were modulated by Nar treatment; arrest appeared to occur at S- and G_2_/M-phases in both colorectal and breast cancer cells. These results are consistent with those reported in other studies using different types of malignancies [31].

Numerous studies have helped scientists appreciate apoptosis as an ideal way to eliminate precancerous and/or cancer cells [37]. However, most cancer cells block apoptosis, which allows malignant cells to survive despite genetic and morphologic transformation. Therefore, searching for agents that can trigger apoptosis in tumor cells has become an attractive strategy in anti-cancer drugs discovery. Here, Nar markedly induced apoptosis in both colorectal and breast cancer cell lines. The results are in line with other several studies that have shown the capability of Nar to stimulate apoptosis in different cancer cell lines [24,31,38,39]. Moreover, emerging evidence from literature has also demonstrated that the anti-proliferative effect of natural products is associated with apoptosis induction [40-42]. Again, the results from this study are in line with these findings. Taken together, dysregulation of the cell cycle mechanism and the induction of cancer cell apoptosis are recognized as an important goal of cancer therapy.

It is known that PI3K and its downstream substrate Akt have a role in apoptosis [43]. The expression of these proteins was examined, identifying dose-dependent reductions of PI3K and phospho-Akt in both colorectal and breast cancer cell lines. Total Akt protein levels remained unchanged throughout the course of the experiment. These results are in agreement with those documented from other studies in other malignancies [42,44].

The PI3K/Akt signaling pathway is influential in the regulation of cell survival due to the activation of anti-apoptotic downstream effectors [45] and phosphorylation-dependent inhibition of pro-apoptotic signals such as Bad, caspase-9, and the family of Forkhead transcription factors [46]. Akt inhibition results in the upregulation of FasL expression in vascular smooth muscle cells and Hela cells [47]. Downregulation of Akt leads to a decrease in the phosphorylation of the endogenous Forkhead factors and its location to the nucleus. It impairs the induction of FasL promoter [46]. In addition, Akt promotes cell survival *via* NF-κB activation [48]. The present study sought to determine the effects of PI3K/Akt on Nar-induced apoptosis. The results showed that Nar down-regulated the PI3K/Akt signaling pathway and that the PI3K/Akt pathway inhibition may increase Nar-induced apoptosis. These data are in accord with those reported in other studies in other types of malignancies [29,42,44]

NF-κB participates in multiple steps in cancer cell resistance to chemical and radiation therapies [18] highlighting its significance as a target in cancer treatment and chemoprevention [49]. We therefore explored this transcription factor as a target for the treatment of human colorectal and breast cancer cells by using Nar. Our results indicate that NF-κB is constitutively active in human breast and colorectal cancer cell lines examined and that Nar down-regulated the nuclear pool, or active form, of NF-κB and suppressed IκBα phosphorylation and the expression of the NF-κB-regulated gene products IκBα, Bcl2 and c-IAP-2. This led to the suppression of proliferation and induction of apoptosis. Nar appears to suppress IKK activation (under investigation) which leads to inhibition of IκBα phosphorylation, as reported in the current study abrogating IκBα degradation by ubiquitin-proteasome pathway.

An imbalance between cell proliferation and cell death due to cell cycle disruption will lead to cancer development. Thus, the cell cycle could serve as a target for anticancer agents to inhibit the uncontrolled proliferation of cancer cells and initiate their apoptosis [50-52]. In the present study, expression of cell cycle-regulatory genes was assessed to understand the underlying molecular mechanisms of Nar anticancer and apoptosis-inducing effects. Human colorectal and breast cancer cells treated with Nar exhibited a marked down-regulation in the mRNA of genes related to cell cycle control, including *Cdk4*, *Cdk6* and *Cdk7*. However, the same treatment resulted in the upregulation of cell cycle-dependent kinase inhibitor genes, *p18*, *p19* and *p21*. These results are consistent with the findings in other types of malignancies [31,42]. Increased levels of p18, p19, p21 and maintenance of the key cell cycle regulatory proteins that counter proliferation signals, eventually leading to apoptosis, are possible mechanisms of colorectal and breast cancer cell death induced by Nar.

Apoptosis is a cell suicide mechanism that is frequently dysregulated in oncogenesis. Generally, apoptosis occurs *via* two fundamental pathways: 1) the mitochondrial or intrinsic pathway and 2) the death receptor or extrinsic pathway [53]. The intrinsic pathway is triggered by the release of mitochondrial proteins, such as cytochrome *c*, which bind to Apaf-1 and procaspase-9 in an ATP-dependent manner to form the apoptosome [54]. The apoptosome activates caspase-9, thereby initiating the apoptotic caspase cascade [55]. Conversely, the extrinsic pathway is initiated by the interaction of ligands with their respective death receptors, sequentially leading to cleavage of the initiator, caspase-8. Active caspase-8 cleaves the executioner procaspase-3, resulting in apoptosis [56]. The apical proteases in the intrinsic and extrinsic pathways are caspase-9 and caspase-8, respectively. Activated caspase-8 and caspase-9 further initiate activation of the caspase cascade, leading to biochemical and morphological changes associated with apoptosis [57]. Caspase-3 is a well-known downstream adaptor caspase that is proteolytically activated by caspase-9 or caspase-8 *via* mitochondrial or cell death receptor signaling pathways [58]. Thus, caspases have been shown to be activated during apoptosis in many cells and play critical roles in both the initiation and execution of apoptosis [59]. To characterize the apoptotic mechanisms induced by Nar, the expression of pro- and anti-apoptotic proteins were assessed. The human colorectal and breast cancer cells exposed to Nar displayed a marked up-regulation in the mRNA expression of pro-apoptotic genes including *Caspases*-*3*, *7*, *8*, and *9*, *Bak*, *AIF* and *Bax*. Meanwhile, the mRNA expression of the anti-apoptotic genes including *Bcl2*, *x-IAP* and *c-IAP-2* was differentially down-regulated in both colorectal and breast cancer cells. These results are in agreement with those findings in other malignancies [29,31,41,42,60].

Numerous studies have demonstrated that the Bcl-2 family significantly regulates apoptosis, either as an activator (Bax) or as an inhibitor (Bcl2) [61]. Bcl2 is a known anti-apoptotic protein that is frequently examined for potential clinical use as a prognostic biomarker in cancer, and its overexpression is associated with resistance to cytotoxic drugs such as cisplatin and 5-fluorouracil. In addition, studies have shown that aberrant expression of this protein facilitates tumor progression [62].

The results presented here demonstrated that Nar-induced apoptosis relates to augmented levels of Bax and down-regulation of Bcl2 inducing mitochondrial dysfunction and leading to apoptosis of colorectal and breast cancer cells.

Bax is an essential mitochondrial-mediated apoptosis activator, as its insertion in the mitochondrial membrane results in the release of cytochrome *c* into the cytosol leading to the activation of caspases and committing the cells to apoptosis [63].

The caspase family, aspartate-specific cysteine proteases, also plays a critical role in regulating apoptosis. Caspase signaling is initiated and propagated by proteolytic autocatalysis and by the cleavage of downstream caspases and substrates such as PARP and lamin A [64]. In particular, caspase-3 is one of the key executioners of apoptosis because it is either partially or totally responsible for the proteolytic cleavage of many key proteins that are important for cell viability [65]. Caspase-9 is an initiator caspase in the apoptotic process, and its function is to activate the effector caspases 6, 7 and 3 [66]. Caspase-independent pathways are involved in mitochondrial dysfunction leading to release of AIF and Endo G from mitochondria and apoptosis [66]. The results from real-time PCR also showed that Nat promoted AIF expression in both colorectal and breast cancer cells, suggesting that Nar-induced apoptosis also involves caspase-independent mechanisms.

The upregulation of pro-apoptotic genes in conjunction with the down-regulation of anti-apoptotic genes in colorectal and breast cancer cells treated with Nar may serve to shift the balance from pro-survival to pro-apoptotic. Nar treatment, thus, may lower the threshold of colorectal and breast cancer cells to pro-death signals, thereby accounting for its anticancer effects. It is well established that agents capable of inducing apoptosis as a mode of cell death are good anticancer candidates [67]. Therefore, Nar is a potential anticancer candidate that is believed to selectively induce apoptosis in cancer cells.

Chemotherapy is one of the main strategies to eliminate residual cancer cells and prevent metastasis after surgery or radiotherapy. The survival period and quality of life of cancer patients have improved with the advent of novel chemotherapeutic agents, such as docetaxel [68]. However, patients continue to suffer various toxic effects including myelosuppression, vomiting and hypersensitivity reactions. Meanwhile, the development of drug resistance continues to threaten patient prognosis [69]. Chemotherapy may also sometimes aggravate cancer progress and lead to patient death [70]. Recently, the concept “to live in harmony with the tumor” has been proposed. This notion has become a new direction in cancer research [71]. In the present study, Nar markedly reduced the apoptotic threshold of both colorectal and breast cancer cells. These results encouraged us to evaluate the potential of Nar to enhance the sensitivity of colorectal and breast cancer cells to DNA-damaging drugs. The results clearly indicated that simultaneous treatment with Nar and DNA-acting drugs exhibited substantially higher cytotoxicity on both colorectal and breast cancer cells compared with a single treatment with a DNA-acting drug. Nar potentiates the chemosensitivity of tested cancer cells in a drug- and cancer-type dependent manner (Figures 6, 7, Table 2). These results are in line with findings in other malignancies [31,72,73].

Thus, Nar has the potential to be a useful adjunct to improve the effectiveness of chemotherapeutic agents in the treatment of human cancers. Nar potentiates the anticancer effects of DNA-acting drugs by activating pro-apoptotic signaling, negating survival signaling, and attenuating their side effects. Testing this strategy with a larger number of cancer cell lines would increase the value of this study. In addition, *in vivo* studies using animal models are necessary to confirm the validity of this combination strategy for the treatment of colorectal and breast cancers and possibly other types of cancers.

## Conclusions

The findings of the present study have important clinical implications. This study demonstrated that Nar induces apoptosis and potentiates the sensitivity of both colorectal and breast cancer cells through multifactorial mechanisms including cell cycle arrest, up-regulation of the expression of pro-apoptotic genes, down-regulation of anti-apoptotic genes and inhibition of pro-survival signaling pathways. Nar therefore displays promise as a pro-apoptotic factor that may benefit colorectal and breast therapy. However, further studies should be conducted in appropriate animal models of cancer, and ultimately, human cancer prevention trials should be performed.
